# Selective organ preservation with neo-adjuvant chemotherapy for the treatment of muscle invasive transitional cell carcinoma of the bladder

**DOI:** 10.1038/bjc.2015.109

**Published:** 2015-04-21

**Authors:** S Hafeez, A Horwich, O Omar, K Mohammed, A Thompson, P Kumar, V Khoo, N Van As, R Eeles, D Dearnaley, R Huddart

**Affiliations:** 1 The Royal Marsden NHS Foundation Trust, London, UK; 2 The Institute of Cancer Research, London, UK

**Keywords:** bladder cancer, transitional cell carcinoma, radiotherapy, chemotherapy, cystectomy, organ preservation

## Abstract

**Background:**

Radiotherapy for muscle invasive bladder cancer (MIBC) aims to offer organ preservation without oncological compromise. Neo-adjuvant chemotherapy provides survival advantage; response may guide patient selection for bladder preservation and identify those most likely to have favourable result with radiotherapy.

**Methods:**

Ninety-four successive patients with T2-T4aN0M0 bladder cancer treated between January 2000 and June 2011 were analysed at the Royal Marsden Hospital. Patients received platinum-based chemotherapy following transurethral resection of bladder tumour; repeat cystoscopy (±biopsy) was performed to guide subsequent management. Responders were treated with radiotherapy. Poor responders were recommended radical cystectomy. Progression-free survival (PFS), disease-specific survival (DSS) and overall survival (OS) were estimated using Kaplan–Meier method; univariate and multivariate analyses were performed using the Cox proportional hazard regression model.

**Results:**

Response assessment was performed in 89 patients. Seventy-eight (88%) demonstrated response; 53 (60%) achieved complete response (CR); 74 responders had radiotherapy; 4 opted for cystectomy. Eleven (12%) demonstrated poor response, 10 received cystectomy. Median survival for CR was 90 months (95% CI 64.7, 115.9) compared with 16 months (95% CI 5.4, 27.4; *P*<0.001) poor responders. On multivariate analysis, only response was associated with significantly improved PFS, OS and DSS. After a median follow-up of 39 months (range 4–127 months), 14 patients (16%) required salvage cystectomy (8 for non-muscle invasive disease, 5 for invasive recurrence, 1 for radiotherapy related toxicity). In all, 82% had an intact bladder at last follow-up after radiotherapy; 67% had an intact bladder at last follow-up or death. Our study is limited by its retrospective nature.

**Conclusions:**

Response to neo-adjuvant chemotherapy is a favourable prognostic indicator and can be used to select patients for radiotherapy allowing bladder preservation in >80% of the selected patients.

Radical cystectomy with pelvic node dissection remains the global standard for the treatment of muscle invasive bladder cancer (MIBC) (Gakis *et al*, 2013). Although advances in continent diversions, nerves sparing procedures and minimally invasive surgical techniques have been made a significant proportion of patients remain unsatisfied over postoperative continence, sexual function and overall quality of life (Hautmann *et al*, 1999; Kessler *et al*, 2005; Pruthi *et al*, 2010). Therefore, in the absence of functional substitutes, it remains important to consider if it is possible to retain native bladder function while maintaining oncological outcomes. Recent bladder preservation studies have demonstrated that this can be achieved using multi-modality treatment with cure rates comparable to contemporary cystectomy series (Coppin *et al*, 1996; Hoskin *et al*, 2010; Efstathiou *et al*, 2012; James *et al*, 2012; Mak *et al*, 2014; Ploussard *et al*, 2014).

Critical to favourable outcome with bladder preservation is appropriate patient selection (Gakis *et al*, 2013). Selective bladder preservation protocols use a predictive marker to aid decision-making regarding definitive treatment. Complete response (CR) to induction treatment is associated with improved survival and has been suggested as a way to help identify individuals who may be particularly suitable for bladder sparing (Splinter *et al*, 1992; Grossman *et al*, 2003; Sternberg *et al*, 2003). Cystectomy is reserved for local recurrence as a radical salvage option with no adverse impact on subsequent survival or incidence of metastatic disease (Rödel *et al*, 2002). Poor response to induction treatment and low chance of cure with bladder preservation proceed directly to radical cystectomy.

The most widely investigated induction strategy employs trans-urethral resection of the bladder tumour (TURBT) and induction chemo-radiotherapy. Cystoscopy is performed after approximately 40 Gy with complete responders receiving a consolidative course of chemo-radiotherapy to a total dose of 64 Gy. Using this technique, approximately 70% of patients will have preserved their own bladders after a median follow-up of 7.7 years (Efstathiou *et al*, 2012). The drawbacks are that all patients will receive some radiation and the gap during the course of radiotherapy to assess response may have adverse radiobiological implications by prolonging overall treatment time theoretically favouring tumour repopulation (Steel, 2002).

Neo-adjuvant cisplatin combination chemotherapy has an absolute survival benefit (approximately 5% at 5 years) irrespective of whether patients then proceed to cystectomy or radiotherapy and is recommended for those who are suitable (Advanced Bladder Cancer Meta-analysis Collaboration, 2003; Advanced Bladder Cancer (ABC) Meta-analysis Collaboration, 2005; Griffiths *et al*, 2011). Selective bladder preservation using response to neo-adjuvant chemotherapy would be an alternative approach but has been less frequently investigated (Sternberg *et al*, 2003).

The only phase 3 study to determine the use of neo-adjuvant chemotherapy alone to guide patient selection for radical radiotherapy closed due to poor recruitment (Paramasivan *et al*, 2011). In the absence of randomised control studies, here we report on our 10-year experience of selective organ preservation with radiotherapy using neo-adjuvant chemotherapy.

## Materials and methods

This analysis is of an institutional-approved protocol identifying patients treated with a selective bladder preservation approach using neo-adjuvant chemotherapy at the Royal Marsden Hospital NHS Foundation Trust between January 2000 and June 2011.

### Patient eligibility

Eligible patients had histological evidence of muscle invasive transitional cell carcinoma (TCC) of the bladder and were staged according to the American Joint Committee on Cancer (Seventh edition) as T2-T4aN0M0. Radiological assessment at baseline was with computer tomography (CT) of the chest, abdomen and pelvis; magnetic resonance imaging (MRI) was the preferred imaging modality to stage the pelvis.

All patients were suitable for platinum-based neo-adjuvant chemotherapy and both radical cystectomy and radical radiotherapy. They were required to have adequate bladder function, absence of uncorrected (unstented) hydronephrosis, adequate renal function for platinum chemotherapy, absence of widespread or distant carcinoma *in situ* (CIS), willingness to undergo neo-adjuvant chemotherapy with no preference for definitive treatment and no objection to long-term endoscopic follow-up if radiotherapy was recommended.

Those participating in the phase 3 randomised control study (SPARE, CRUK/07/011) were not included; their outcomes have been presented separately (Huddart *et al*, 2012).

Modified Charlson–Deyo score (measure of comorbidity across multiple organ sites, captured using International Classification of Diseases, Ninth revision, Clinical Modification codes) was retrospectively calculated for each patient excluding his or her diagnosis of MIBC (Deyo *et al*, 1992). The presence of hydronephrosis and hydroureter at presentation was also recorded, including whether ureteric stenting was performed.

### Treatment

Patients were treated with initial TURBT. In accordance with clinical guidelines, safe thorough TURBT was recommended. Following TURBT, patients received 3–4 cycles of neo-adjuvant chemotherapy. This was methotrexate, vinblastine, Adriamycin (doxorubicin) and cisplatin (accelerated MVAC, also known as dose dense MVAC; 14-day cycle with granulocyte colony-stimulating factor, GCSF) or gemcitabine with cisplatin (GC); alternative platinum combination chemotherapy were also permitted (Table 1).

Assessment of response was made with repeat cystoscopy and tumour site biopsy (where possible) 3 weeks after neo-adjuvant chemotherapy. Although endoscopic assessment was preferred as the gold standard in occasional circumstances, radiological evaluation (CT and/or MRI) was also necessary in order to support clinical decision-making.

CR was defined as lack of residual tumour. If tumour site biopsy identified no residual disease, this was deemed to be a pathological CR; if no disease was visible at endoscopy to biopsy, this was deemed to be a complete CR. Partial response (PR) was defined as either pathologically down staging to pTa, pT1, pTis or radiological evidence of response. Poor response was defined as residual muscle invasive disease on biopsy (pT2) or radiological evidence of disease progression. Poor responders were recommended immediate radical cystectomy; responders proceeded to radical radiotherapy to the bladder (64–68 Gy in 2 Gy per fraction). After 2004, concurrent chemo-radiotherapy was introduced (mitomycin and 5-flurouracil, see Table 1).

### Follow-up

Those patients treated with radical radiotherapy were followed up on a cystoscopic surveillance schedule. Patients with recurrence were promptly considered for either intra-vesical therapy or radical salvage cystectomy depending on the nature of the local recurrence.

### Outcomes

Overall survival (OS) and progression-free survival (PFS) were defined as time from the start of chemotherapy to death from any cause and interval from chemotherapy initiation to relapse (radiological or clinical) or death, respectively. Disease-specific survival (DSS) was defined as surviving the protocol treatment and having no evidence of distant metastases, nodal recurrence or local recurrence within the bladder that could not be salvaged with curative intent.

Surviving patients, PFS and those lost to follow-up were censored at the last assessment date. Median time to PFS, OS and DSS were estimated using Kaplan–Meier method. Univariate and multivariate analysis were performed using the Cox proportional hazard regression model. All variables with *P*-value <0.20 were used in the forward stepwise method for the multivariate model. Chi-squared analyses were performed to evaluate the effect of covariates on achieving a response to neo-adjuvant chemotherapy. A *P*-value <0.05 was considered statistically significant. All analyses were carried out using SPSS v.22 (IBM, Chicago, IL, USA).

## Results

Between January 2000 and June 2011, 94 successive patients with T2-T4aN0M0 bladder TCC underwent neo-adjuvant chemotherapy with the intention to be treated with a selective bladder preservation approach. Median age was 65 years (range 34–83 years). Patient characteristics are shown in Table 2.

Deviation from standard cisplatin-based neo-adjuvant chemotherapy regime occurred in 12 patients who received carboplatin and gemcitabine (5 had hydronephrosis at diagnosis; 4 had renal impairment with no hydronephrosis; 1 patient had preexisting tinnitus; in 1 patient age was cited, he was 83 years old; and in 1 patient the reason was unrecorded). Summary of chemotherapy regime response rate is presented in Table 3.

Postchemotherapy response assessment was performed in 89 patients. Two patients declined assessment expressing a preference for radiotherapy (1 patient) or surgery (1 patient); 2 patients died before their assessment, 1 from confirmed pulmonary embolism at postmortem following 2 cycles of accelerated MVAC chemotherapy, the other from non-neutropenic lower respiratory tract infection complicated by pulmonary embolism after 3 cycles of accelerated MVAC. One patient proceeded directly to radiotherapy because he had become unfit for radical surgery following an episode of neutropenic sepsis after 1 cycle of carboplatin and gemcitabine chemotherapy.

### Response assessment and definitive treatment

Endoscopic assessment of response was made in 76 patients. For 13 patients, response assessment was made on radiology alone.

Seventy-eight out of 89 (88%) assessable patients demonstrated a favourable response to neo-adjuvant chemotherapy. Fifty-three patients (60%) achieved CR, including 44 (49%) confirmed with negative tumour bed biopsy. Twenty-five patients (28%) demonstrated PR. Eleven (12%) patients demonstrated poor response.

**Figure 1:**
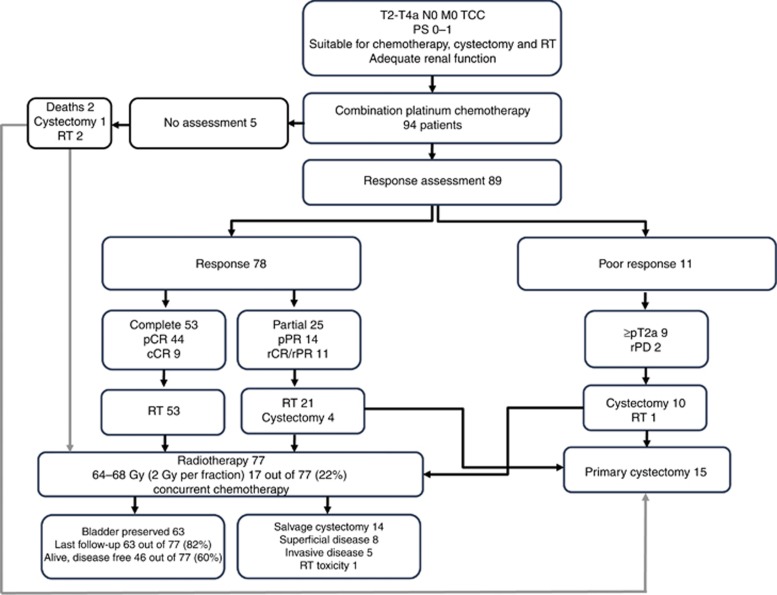
**Patient study flow.** cCR=clinical complete response; pCR=pathological complete response; PD=disease progression; pPR=pathological partial response; PS=performance status; rCR=radiological complete response; rPR=radiological partial response; RT=radiotherapy

Seventy-four patients who demonstrated response went on to have radical radiotherapy; 4 opted for cystectomy. All 11 patients demonstrating poor response were recommended radical cystectomy; however, 1 patient opted for radical radiotherapy. The patient study flow is presented in Figure 1.

On chi-squared analysis, there was no significant association between tumour and patient characteristics and response to neo-adjuvant chemotherapy apart from the presence of hydronephrosis, where the trend demonstrated that those with hydronephrosis had poor response (*P*=0.037).

### Outcome and survival

After a median follow-up of 39 months (4–127 months), 52 (55%) patients were alive and disease free, and 36 (38%) had died. Median PFS, OS and DSS were 36.9 months (95% CI 27.9, 46.0 months), 90.3 months (95% CI 42.2, 138.4 months) and 112.4 months (95% CI 69.5, 155.3 months), respectively. Table 4 summarises the outcome grouped by response to neo-adjuvant chemotherapy and definitive treatment.

Following radical cystectomy, 50% (8 out of 15) experienced disease recurrence (2 patients with pelvic recurrences and 6 with distant metastases). Following radical radiotherapy, 47% (36 out of 77) patients experienced disease recurrence. In all, 70% (25 out of 36) of first recurrences after radiotherapy occurred within the bladder. Also, 25% (19 out of 77) developed non-muscle invasive bladder cancer; 10 were treated with intra-vesical therapy alone, 8 required cystectomy (2 for intra-vesical treatment failure; 6 as primary salvage), and one patient declined further treatment following TURBT. A total of 8% (6 out of 77) developed local invasive recurrence, 5 proceeded to salvage cystectomy but 1 patient was unfit for radical salvage. No intra-vesical recurrences occurred in those surviving >5 years after radiotherapy. One recurrence occurred 5 years after radiotherapy, this was metastatic in nature.

Seventeen of the 77 radiotherapy patients received concurrent chemotherapy. In all, 41% (7 out of 17) went on to experience disease recurrence. Also, 24% (4 out of 17) of first recurrences occurred within the bladder (3 with non-muscle invasive bladder cancers and 1 with an invasive recurrence). Sub group univariate analysis demonstrated that concurrent chemotherapy did not reach statistical significance for PFS (HR 1.1 95% CI 0.5, 2.3: *P*=0.76), OS (HR 1.0 95% CI 0.4, 2.8: *P*=0.93) or DSS (HR 1.3 95% CI 0.4, 4.1: *P*=0.65).

During the follow-up period, only one patient required cystectomy because of radiotherapy-related urinary toxicity. Medium time to salvage cystectomy was 19.6 months (range 9.8–82.7months). Bladder preservation rate was 82% (63 out of 77) of those undergoing radical radiotherapy and in 67% (63 out of 94) of the total patient population. Of the 20 patients whose follow-up has reached ⩾5 years, bladder preservation rate was 65% (11 out of 17) in the radiotherapy group and 55% (11 out of 20) in all survivors beyond 5 years.

Among patients achieving a CR after neo-adjuvant chemotherapy, 55% patients developed no further disease recurrence compared with 18% with poor response following chemotherapy. Median survival for complete responders was significantly better than poor responders, 90.3 months (95% CI 64.7, 115.9) compared with 16.4 months (95% CI 5.4, 27.4) (*P*<0.001).

**Figure 2:**
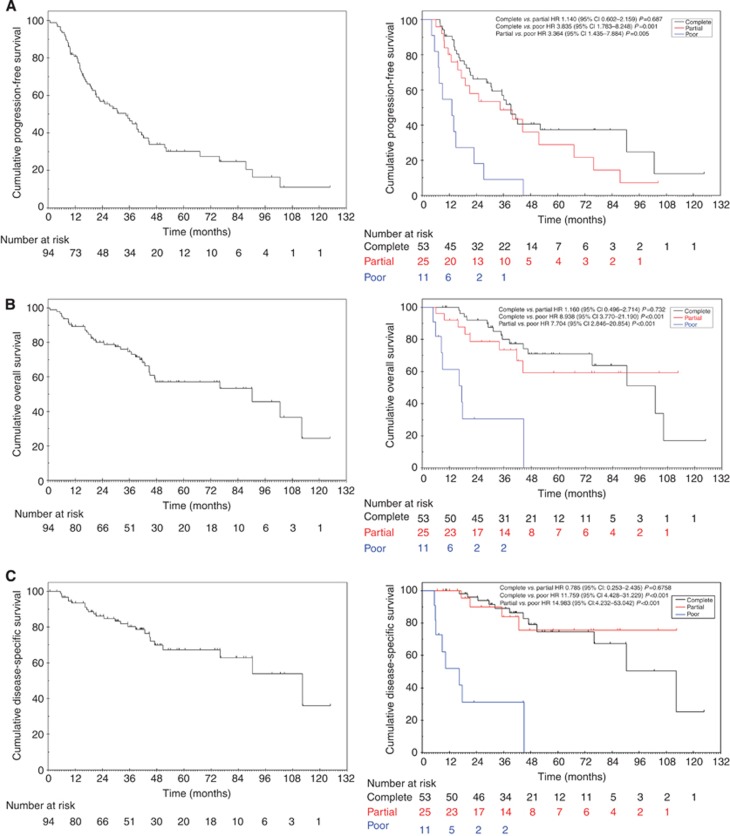
**Kaplan–Meier plots for rates of (**A**) progression-free survival, (**B**) overall survival and (**C**) disease-specific survival for all patients and stratified by response to neo-adjuvant chemotherapy.**

On univariate analysis, poor responders to neo-adjuvant chemotherapy had significantly worse outcome in terms of PFS, OS and DSS compared with those demonstrating any response (CR or PR) (Table 5). No significant difference in PFS, OS and DSS was seen between CR and PR (Figure 2).

The neo-adjuvant chemotherapy regime also significantly impacted on outcome. Those receiving gemcitabine–carboplatin had worse OS compared with gemcitabine–cisplatin (HR 3.6 95% CI 1.4, 9.6: *P*=0.01) and DSS (HR 2.8 95% CI 0.95, 8.29: *P*=0.06). No significant difference was seen between gemcitabine–cisplatin and accelerated MVAC in OS (HR 1.2 95% CI 0.6, 2.8: *P*=0.61) or DSS (HR 0.6 95% CI 0.2, 1.48: *P*=0.24).

On univariate analysis, the presence of hydronephrosis was associated with decreased PFS (HR 2.5 95% CI 1.1, 5.7: *P*=0.02), OS (HR 4.9 95% CI 2.0, 12.3: *P*=0.001) and DSS (HR 5.1 95% CI 1.7, 15.8: *P*=0.005).

On multivariate analysis of tumour and patient characteristics, only response to neo-adjuvant chemotherapy maintained statistical significance for PFS, OS and DSS when CR was compared with poor response and when PR was compared with poor response (Table 6).

## Discussion

Our long-term results support the use of neo-adjuvant chemotherapy response to guide patient selection for radical radiotherapy. This approach successfully allows organ preservation in approximately 70% of all patients and >80% of patients receiving radiotherapy with survival comparable to recent surgical series (Gakis *et al*, 2013).

Following radiotherapy, the majority (70%) of relapses occurred within the bladder and were amenable to subsequent salvage treatment. Encouragingly, only 6 (8%) patients have developed invasive disease. Most (76%) were non-muscle invasive recurrences, over half of which were successfully managed with TURBT and intravesical therapy, so still allowing patients to preserve functional bladders while maintaining long-term cure. Indeed after intravesical treatmen,t only 2 patients have had further relapse requiring salvage cystectomy. This pattern of relapse does raise the issue as to whether maintenance intra-vesical therapy should be considered in line with the management of patients with pT1G3 disease. It also means that life-long surveillance is mandated in order that prompt salvage can be implemented. These patients therefore have to be committed to regular cystoscopies, as this remains the gold standard for evaluation (Gakis *et al*, 2013). All intra-vesical relapses within our patient population, however, occurred within 5 years of completing radiotherapy; further evidence and longer follow-up may help to inform the actual intensity of cystoscopic surveillance beyond 5 years.

It has been suggested that the presence of CIS on original biopsy should be a contraindication to bladder preservation. However, on the limited data we have available we find no evidence to support this assertion.

Although no formal assessment of bladder function or quality of life was performed, only one patient required cystectomy for radiotherapy-related urinary toxicity. Previous work corroborates acceptable genito-urinary and gastro-intestinal toxicity following radiotherapy and chemo-radiotherapy (Zietman *et al*, 2003; Efstathiou *et al*, 2009; Efstathiou *et al*, 2012; James *et al*, 2012).

The historical nature of the cohort meant that the majority of patients received radiotherapy alone; only 22% received concurrent chemo-radiotherapy. Phase 3 evidence supports he use of mitomycin and 5-flurouracil to improve local–regional disease-free survival compared with radiotherapy alone (HR 0.68 95% CI, 0.48 to 0.96; *P*=0.03) (James *et al*, 2012). Thus the universal use of these or other radiosensitising agents could further improve the local control rates reported here (Hoskin *et al*, 2010; Choudhury *et al*, 2011; Gakis *et al*, 2013; Mitin *et al*, 2013).

Sixty-seven percent of local recurrences after radiotherapy occur at the original bladder tumour site, supporting the likelihood of persistent occult disease (Zietman *et al*, 2001). The radiation dose–response relationship of these tumours means that higher radiation doses targeted to the bladder tumour may offer further opportunity to improve local control (Huddart *et al*, 2014). Partial cystectomy as an alternative organ-sparing approach does not offer universal substitution for radiotherapy as only 5% of MIBC meet the stringent criteria necessary to ensure acceptable local control rates, including bladder dome lesions where a minimum 2-cm margin can be removed without compromise to continence or bladder capacity (Gakis *et al*, 2013).

The rate of pelvic nodal relapse following radiotherapy is low, consistent with other reported series (James *et al*, 2012; Mak *et al*, 2014). Historically, bladder radiotherapy used large margins to capture geometric uncertainty of filling and motion during treatment and is likely therefore to have encompassed at-risk pelvic nodal groups to some extent, potentially delivering enough dose to sterilise disease. The recent implementation of more accurate radiotherapy (image guided and intensity modulated) has lent itself to margin reduction as certainty in dose delivered to target has improved; in turn, this could impact on incidental pelvic nodal irradiation and so future nodal relapse rates will need to be monitored. In practice, however, the question of whether pelvic irradiation in bladder cancer should be used at all remains unclear and a point of on-going debate.

Response to neo-adjuvant chemotherapy was identified as an important prognostic indicator and was the only significant predictor of survival on multivariate analysis of this cohort. No difference in outcome was seen between those demonstrating CR or PR to chemotherapy.

Poor response to neo-adjuvant chemotherapy was associated with significantly worse PFS and OS despite radical treatment with cystectomy. The majority of poor responding patients (73%, 8 out of 11) subsequently died from metastatic disease. This is consistent with earlier series reflecting less favourable outcome for poor responders to induction therapy, with a reported 5-year DFS of 20% and >40% of patients unfortunately developing metastases within 2 years (Rödel *et al*, 2002). Intensification of systemic treatment to reduce metastatic relapse should therefore be an important consideration. The molecular characterisation of MIBC opens the door to potential personalised systemic therapy with targeted agents for those unlikely to respond to conventional platinum therapy (Cancer Genome Atlas Research Network, 2014; Choi *et al*, 2014). The poor outcome at present, however, raises the question about the standard conventional approach to offer these patients cystectomy given that systemic disease control currently remains an issue for the majority of poor responders.

Current selective bladder preservation protocols are reliant on response to induction therapy to inform definitive treatment. This approach has a number of limitations. Chemotherapy is not without significant toxicity and potential mortality. Those who do not respond are exposed to morbidity with no certain benefit and a delay in effective treatment. The challenge remains how best to identify these patients and their tumour characteristics at diagnosis to determine individual benefit from chemotherapy and likelihood of cure with radical radiotherapy.

Many groups have performed single-marker studies but no biomarker is used routinely in the clinic to select patients for treatment. For example, high MRE11 expression specifically predicts improved outcome with radiotherapy, demonstrating 16% improvement in 3-year cancer-specific survival (CSS) compared with high MRE11 expression treated with cystectomy (69.9% *vs* 53.8% 3-year CSS, *P*=0.021) (Choudhury *et al*, 2010). It is envisaged that MRE11 expression could be used to guide future consultations with patients about bladder radiotherapy, but prospective studies and standardised laboratory testing are needed before this transition to clinical practice can occur.

Previous studies have identified T stage as a strong predictor of outcome (Efstathiou *et al*, 2012). Our multivariate analysis did not reflect this possibly because during the 10-year assessment period routine local staging changed from CT to MRI assessment and additionally relatively small numbers of patients had advanced stage disease. The majority (approximately 75%) of patients were staged as T2 disease at presentation. Although this is consistent with other bladder radiotherapy cohorts, the known discordance between clinical and pathological staging is likely to have further confounded stratification by stage (Efstathiou *et al*, 2012; James *et al*, 2012; Mak *et al*, 2014). The presence of hydronephrosis was associated with worse PFS, OS and DSS on univariate analysis and may have been proxy of advanced local disease. Hydronephrosis was associated with poor response; again suggestive of high T stage and larger tumours, but these patients were also more likely to receive carboplatin chemotherapy (56% compared with 8% with no hydronephrosis).

Completeness of TURBT has also been reported as an important predictor of outcome, but this was difficult to verify retrospectively in our cohort (Rödel *et al*, 2002; Efstathiou *et al*, 2012). Incomplete TURBT reflects the presence of larger tumours and is a surrogate measure of advanced T stage.

Although the numbers are small, those who received carboplatin had a poorer OS. Though this is consistent with the evidence that carboplatin is inferior to cisplatin-containing chemotherapy regimes in both the neo-adjuvant and metastatic setting, it could be due to confounding factors leading to the decision to use carboplatin-based treatment (Advanced Bladder Cancer Meta-analysis Collaboration, 2003; Advanced Bladder Cancer (ABC) Meta-analysis Collaboration, 2005; Gakis *et al*, 2013).

As pathological CR can be achieved with chemotherapy and TURBT, the question whether further treatment is necessary for these patients arises. The phase II SWOG study (S0219) demonstrated no difference in survival between complete responders who went on to have cystectomy or those who had close endoscopic follow-up (70% *vs* 76% respectively). Adopting a strict surveillance protocol after CR with radical treatment offered promptly at progression did not adversely affect OS (deVere White *et al*, 2009). Larger randomised studies would be necessary before those achieving a good response or CR to induction could have conventional radical treatment confidently deferred because their cancer cure had been achieved with induction alone.

## Conclusion

Response to neo-adjuvant chemotherapy is a favourable prognostic indicator and can be used to select patients for radiotherapy allowing bladder preservation in approximately 70% of patients with survival comparable to recent surgical series.

## Figures and Tables

**Table 1:** Chemotherapy regimes used

**Neo-adjuvant chemotherapy regime**	**Drug**				
**Accelerated MVAC**		**Day 1**	**Day 2**	**Days 4–11**	**14-Day cycle**
	Methotrexate 30 mg m^−2^ i.v.	•			
	Vinblastine 3 mg m^−2^ i.v.		•		
	Doxorubicin 30 mg m^−2^ i.v.		•		
	Cisplatin 70 mg m^−2^ i.v.		•		
	GCSF s.c.			•	
**Gemcitabine–cisplatin**		**Day 1**	**Day 8**		**21-Day cycle**
	Gemcitabine 1000 mg m^−2^ i.v.	•	•		
	Cisplatin 70 mg m^−2^ i.v.	•			
**Gemcitabine–carboplatin**		**Day 1**	**Day 8**		**21-Day cycle**
	Gemcitabine 1000 mg m^−2^ i.v.	•	•		
	Carboplatin AUC 4.5	•			
**Concurrent chemotherapy**					
**MMC–5FU**		**Day 1**	**Days 2–5**	**Days 16–20**	
	Mitomycin C 12 mg m^−2^ i.v.	•			
	5FU 500 mg m^−2^ i.v.	•	•	•	

Abbreviations: AUC=area under the curve; GCSF=granulocyte colony-stimulating factor; i.v.=intravenous; MMC=mitomycin C; MVAC=methotrexate, vinblastine, Adriamycin and cisplatin; s.c.=subcutaneous; 5FU=5-flurouracil. Bold dots indicate drug administered.

**Table 2:** Patient characteristics

Age^a^	Median 65 years (range 34–83)
**Gender**	
Male	82
Female	12
**Stage of primary**	
T2	70
T3	18
T4	6
**Grade**	
Intermediate (grade 2)	2
High (grade 3)	92
**Presence of adjacent CIS at diagnosis**	
Yes	23
No	31
Unable to assess	1
No comment made	35
Pathology not available	4
**Charlson comorbidity index**	
0	65
1	14
2	9
⩾3	6
**Hydronephrosis/hydroureter at presentation**	
Absent	85
Present	9 (4 received ureteric stenting)
**Chemotherapy schedule**	
Gemcitabine–cisplatin	43
Accelerated MVAC	39
Gemcitabine–carboplatin	12
**Number of cycles**	Median 3 cycles (range 1–5)
1	1
2	3
3	73
4	16
5	1
**Assessment of response**	
Cystoscopy and biopsy	67
Cystoscopy alone	9
Radiology alone	13
Not assessed	5

Abbreviations: CIS=carcinoma *in situ*; MVAC=methotrexate, vinblastine, Adriamycin and cisplatin.

a 33 patients were ⩾70years; 15 patients were ⩾75years.

**Table 3:** Summary of chemotherapy regime response rate

			**Favourable response of those assessed (%)**		
**Chemotherapy regime**	**Total**	**Not assessed**	**Complete**	**Partial**	**Poor response of those assessed (%)**
All	94	5	53 (60%)	25 (28%)	11 (12%)
Gemcitabine–cisplatin	43	0	29 (67.4%)	10 (23.3%)	4 (9.3%)
Accelerated MVAC	39	3	20 (55.6%)	12 (33.3%)	4 (11.1%)
Gemcitabine–carboplatin	12	2	4 (40%)	3 (30%)	3 (25%)

Abbreviation: MVAC=methotrexate, vinblastine, Adriamycin and cisplatin.

**Table 4:** Status at last follow-up

	**Treatment response to neo-adjuvant chemotherapy**			**Definitive treatment**^a^	
	**Complete**	**Partial**	**Poor**	**Radiotherapy**	**Cystectomy**
**Alive**	37/53	17/25	2/11	52/77	6/15
Disease free	31	17	2	46	6
Localised disease (bladder)	4	—	—	4	—
Local regional disease (pelvis)	1	—	—	1	—
Metastases	1	—	—	1	—
**Dead**	16/53	8/25	9/11	25/77	9/15
Metastases	10	4	8	15	8
Other malignancy	4	1	—	5	—
Other causes	2	1	1	3	1
Unknown	—	2	—	2	—

a All patients (92) who received definitive treatment, including 3 in whom response assessment was not made.

**Table 5:** Univariate analyses for survival end points

**Variable**	**Events (*****n*****=55)**	**Median time, months (95% CI)**	**HR (95% CI)**	***P*****-value**
**Progression-free survival**				
**Age:**				
⩽65	31	37.2 (25.0–49.9)	1	0.81
>65	24	36.2 (13.6–58.7)	1.07 (0.62–1.84)	
**Gender**				
Female	5	Not estimable^a^	1	0.37
Male	50	36.9 (24.4–49.5)	1.53 (0.60–3.85)	
T stage				
T2	39	39.4 (27.1–51.7)	1	0.53
T3	12	34.6 (13.2–56.0)	1.28 (0.67–2.45)	
T4	4	14.1 (0–36.1)	1.64 (0.58–4.61)	
**Chemotherapy regime**				
Gemcitabine–cisplatin	23	37.2 (25.6–48.8)	1	0.51
Gemcitabine–carboplatin	7	19.6 (12.4–26.8)	1.38 (0.59–3.23)	
Acc-MVAC	25	40.5 (30.2–50.8)	0.84 (0.47–1.51)	
**Response**				
Complete	29	40.5 (34.9–46.2)	1	**0.002**
Partial	14	40.0 (14.6–65.4)	1.14 (0.60–2.16)	0.69
Poor	9	14.0 (6.5–21.6)	3.84 (1.78–8.25)	0.001
**Number of cycles**				
2–3	44	39.4 (33.5–45.2)	1	0.38
4–5	11	25.1 (15.9–34.3)	1.36 (0.69–2.66)	
**Presence of CIS at diagnosis**^b^				
CIS	12	39.4 (5.3–73.5)	1	0.54
No CIS	20	30.8 (13.1–48.4)	1.25 (0.61–2.54)	
**Hydronephrosis**				
No	48	39.4 (32.7–46.0)	1	**0.02**
Yes	7	17.3 (11.9–22.7)	2.54 (1.13–5.70)	
**Charlson score**				
0	41	36.9 (19.3–54.6)	1	0.41
1	6	39.4 (30.5–48.3)	0.60 (0.25–1.41)	
⩾2	8	40.0 (16.1–63.9)	1.20 (0.55–2.58)	

**Variable**	**Events (*****n*****=36)**	**Median time, months (95% CI)**	**HR (95% CI)**	***P*****-value**
**Overall survival**				
**Age**				
⩽65	19	102.7 (31.5–173.8)	1	0.24
>65	17	76.0 (33.0–119.0)	1.51 (0.76–3.00)	
**Gender**				
Female	2	Not estimable^a^	1	0.19
Male	34	90.3 (34.0–146.6)	2.58 (0.62–10.82)	
**T stage**				
T2	23	102.7 (51.9–153.5)	1	0.10
T3	10	40.5 (13.0–68.1)	2.21 (1.04–4.68)	
T4	3	44.7 (0–100.0)	1.976 (0.581–6.715)	
**Chemotherapy regime**				
Gemcitabine–cisplatin	10	Not estimable^a^	1	**0.025**
Gemcitabine–carboplatin	7	32.1 (14.6–49.7)	3.61 (1.37–9.55)	0.01
Acc-MVAC	19	90.3 (59.1–121.5)	1.23 (0.55–2.77)	0.61
**Response**				
Complete	16	90.3 (64.7–115.9)	1	**<0.001**
Partial	8	Not estimable^a^	1.16 (0.5–2.71)	0.73
Poor	9	16.4 (5.41–27.4)	8.94 (3.77–21.19)	<0.001
**Number of cycles**				
2–3	30	90.3 (42.2–138.4)	1	0.62
4–5	6	41.8 (29.6–53.9)	1.28 (0.51–3.10)	
**Presence of CIS at diagnosis**^b^				
CIS	9	Not estimable^a^	1	0.74
No CIS	13	Not estimable^a^	1.106 (0.49–2.76)	
**Hydronephrosis**				
No	30	90.3 (62.2–118.4)	1	**0.001**
Yes	6	29.6 (5.7–53.6)	4.90 (1.95–12.32)	
**Charlson score**				
0	41	90.3 (28.6–152.0)	1	0.342
1	6	Not estimable^a^	0.44 (0.13–1.43)	
⩾2	8	47.1 (25.3–69.0)	1.20 (0.45–3.15)	

**Variable**	**Events (*****n*****=25)**	**Median time, months (95% CI)**	**HR (95% CI)**	***P*****-value**
**Disease-specific survival**				
**Age**				
⩽65	16	112.4 (73.1–151.6)	1	0.92
>65	9	76.0 (not estimable^a^)	0.85 (0.40–2.14)	
**Gender**				
Female	2	Not estimable^a^	1	0.44
Male	23	112.4 (74.6–150.1)	1.78 (0.42–7.60)	
**T stage**				
T2	16	112.4 (50.9–173.9)	1	0.19
T3	6	Not estimable^a^	1.94 (0.75–5.01)	
T4	3	90.3 (not estimable^a^)	2.58 (0.74–8.98)	
**Chemotherapy regime**				
Gemcitabine–cisplatin	10	Not estimable^a^	1	**0.028**
Gemcitabine–carboplatin	5	32.1 (not estimable^a^)	2.80 (0.95–8.29)	0.06
Acc-MVAC	10	112.4 (73.2–151.6)	0.55 (0.21–1.48)	0.24
**Response**				
Complete	12	112.4 (79.1–145.7)	1	**<0.001**
Partial	4	Not estimable^a^	0.79 (0.25–2.44)	0.68
Poor	8	16.4 (5.41–27.4)	11.76 (4.43–31.23)	<0.001
**Number of cycles**				
2–3	20	112.4 (73.4–151.5)	1	0.37
4–5	5	Not estimable^a^	1.59 (0.58–4.39)	
**Presence of CIS at diagnosis**^b^, ***n*=17**				
CIS	8	90.3 (27.5–153.1)	1	0.62
No CIS	9	112.4 (not estimable^a^)	0.77 (0.28–2.14)	
**Hydronephrosis**				
No	21	112.4 (73.4–151.4)	1	0.005
Yes	4	24.6 (3.5–45.7)	5.11 (1.65–15.81)	
**Charlson score**				
0	19	112.4 (76.6–148.2)	1	0.36
1	2	Not estimable^a^	0.42 (0.10–1.82)	
⩾2	4	47.1 (9.0–85.3)	1.47 (0.49–4.44)	

Abbreviations: Acc-MVAC=methotrexate, vinblastine, doxorubicin and cisplatin given over 2 weeks with GCSF support (granulocyte colony-stimulating factor); CI=confidence interval; CIS=carcinoma *in situ*; HR=hazard ratio.

a Not possible to estimate, as cumulative survival curve does not fall below 50% and the groups' survival curve did not reach median.

b Missing information regarding the presence or absence of CIS.

**Table 6:** Multivariate analyses for survival end points

	**Progression-free survival**		**Overall survival**		**Disease-specific survival**	
**Variable**^a^	**HR (95% CI)**	***P*****-value**	**HR (95% CI)**	***P*****-value**	**HR (95% CI)**	***P*****-value**
**Response**		0.002		<0.001		0.001
Complete	1		1		1	
Partial	1.14 (0.60–2.16)	0.687	1.16 (0.50–2.71)	0.732	0.79 (0.25–2.44)	0.675
Poor	3.84 (1.78–8.25)	0.001	8.938 (3.77–21.19)	<0.001	11.76 (4.43–31.23)	<0.001

Abbreviations: CI=confidence interval; HR=hazard ratio.

a All variables with *P*-value <0.20 from univariate analysis were used in the forward stepwise method for the multivariate model.
