# Selective organ preservation with neo-adjuvant chemotherapy for the treatment of muscle invasive transitional cell carcinoma of the bladder

**DOI:** 10.1038/bjc.2016.132

**Published:** 2016-05-26

**Authors:** S Hafeez, A Horwich, O Omar, K Mohammed, A Thompson, P Kumar, V Khoo, N Van As, R Eeles, D Dearnaley, R Huddart

**Correction to:** *British Journal of Cancer* (2015) **112,** 1626–1635; doi:10.1038/bjc.2015.109; published online 21 April 2015

**Updated online 26 May 2016:** This article was originally published under a CC BY-NC-SA 4.0 license, but has now been made available under a CC BY 4.0 license. The PDF and HTML versions of the paper have been modified accordingly.

